# Promoting Men’s Health With the “Don’t Change Much” e-Program

**DOI:** 10.1177/15579883211001189

**Published:** 2021-03-20

**Authors:** John L. Oliffe, Nick Black, Jeffrey Yiu, Ryan Flannigan, Wayne Hartrick, S. Larry Goldenberg

**Affiliations:** 1 School of Nursing, University of British Columbia, Vancouver, BC, Canada; 2 Department of Nursing, University of Melbourne, Victoria, Australia; 3 Intensions Consulting, Vancouver, BC, Canada; 4 Department of Urology, Male Reproduction & Microsurgery, Weill Cornell Medicine, New York, NY, USA; 5 Department of Urologic Sciences, University of British Columbia, Vancouver, BC, Canada; 6 Canadian Men’s Health Foundation, Vancouver, BC, Canada

**Keywords:** Men’s health behaviors, men’s health promotion, men’s e-health, health behavior change

## Abstract

Men’s e-health promotion programs can offer end-user anonymity and autonomy that provide avenues for supporting positive health behavior change. The twofold purpose of the current study was to use a benchmark cohort as a reference group to: (1) describe associations between men’s usage levels of the e-health program Don’t Change Much (DCM) and their recent and intended health behavior changes, and (2) report an exploratory analysis of the moderating effects of demographic variables on the associations between DCM users and their recent and intended health behavior changes. Based on self-report, DCM users were classified into limited (*n* = 613, 34.7%), low (*n* = 826, 46.8%), and high (*n* = 327, 18.5%) exposure groups. Compared with the benchmark cohort, DCM high-exposure respondents had significantly increased odds for eight of the nine recent behavior changes, with the largest effect size observed for “Made an effort to sit less and walk more” (odds ratio [OR] 2.996, 95% CI [2.347, 3.826]). Eight of the nine intended health behavior changes in the DCM high-exposure group had significantly increased odds compared to the benchmark cohort, with “Reduce stress level” (OR 3.428, 95% CI [2.643, 4.447]) having the largest effect size. Significantly greater total numbers of recent (*F*(12, 2850) = 29.32; *p* = .001; R^2^ = .086) and intended health behavior changes (*F*(12, 2850) = 34.59; *p* = .001; R^2^ = 0.100) were observed among high exposure respondents while adjusting for demographics. Younger age, being employed, and household income <$120,000 had an enhancing moderator effect on DCM users’ number of intended behavior changes.

Many health promotion programs have focused on reducing male risk factors such as unhealthy diets, substance use, smoking, and sedentary lifestyles (Lahoud & Franco, 2016). Investigations of the challenges for engaging men with self-health have highlighted the need for gender–sensitized health promotion programs (Oliffe et al., 2020a; Oliffe et al., 2020b; Soprovich et al., 2020). While the delivery of men’s health promotion programs is diverse, the e-health sector has grown significantly during the 2000s (Fogarty et al., 2017). By offering end-user anonymity and autonomy for selecting specific content, men’s e-health promotion programs can provide important avenues to augment and connect traditional services and aid men’s behavior changes and health promotion practices (Robinson & Robertson, 2010). Within the men’s e-health context, there is much debate about program effectiveness as well as broader concerns regarding the relative reach of interventions for specific male subgroups.

## Background

Men’s e-health promotion programs have grown rapidly to address diverse contexts and deliver tailored information through an array of platforms (i.e., web, social media, email, etc.). While men’s health issues including prostate cancer (Bender et al., 2019), HIV prevention (Klein et al., 2017), weight loss (Young & Morgan, 2018), and health screening (Teo et al., 2019) have attracted e-health promotion programs, there is much debate about the effectiveness of these interventions. This relates, in part, to the predominance of needs analyses and acceptability studies wherein the feasibility and/or design of e-health programs are informed by potential male end-users. For example, a study of men with prostate cancer indicated 65% (*n* = 784) of respondents used the internet as a source of prostate cancer information, and that this subgroup was more likely to have unmet supportive care needs (Bender et al., 2019). These findings led the authors to recommend examining men’s e-health literacy as the next step to tailoring internet-based prostate cancer information (Bender et al., 2019). Differentiating these needs analyses, men’s e-health programs have also been pilot-tested with a view to adjusting content and delivery based on end-user and expert feedback. Examples include acceptability testing of a mobile application to improve men’s health screening uptake, which showed positive utility and usability amongst end-users (Teo et al., 2019). Some formal evaluations have also linked men’s behavior change to their use of specific e-health programs. A men’s e-health weight loss program was positively evaluated in a pre-post study design wherein short-term improvements in the mental health of overweight and obese men were reported (Young & Morgan, 2018). Man Central (a web and mobile phone intervention for men with depression) reported significant improvements in depression symptoms, depression risk, externalizing symptoms, and work and social functioning among end-users based on data collected through repeated measures from a single respondent group (Fogarty et al, 2017). A quasi-experimental, two-arm study by Klein et al. (2017) evaluated “Real Talk” (an e-health harm reduction intervention targeting Black men who have sex with men) and reported increased end-user HIV knowledge, though no significant differences for condom use or other risk reduction practices compared to the control group were found. These program reports and evaluations, and many other men’s e-health promotion studies, provide important insights; however, the findings and the men’s e-health field more broadly are limited by small sample sizes, cross-sectional study designs, and/or a lack of control groups (Forbes et al., 2019).

There has been research investigating the relative reach of men’s e-health promotion programs with a focus on distilling how social determinants of health influence men’s access, usage, and potential benefits (Francis, 2019; Nguyen et al., 2019). For example, low income (Dzinamarira et al., 2020) and homeless (Calvo et al., 2019) men have been highlighted as experiencing health inequities, which depending on the e-health content and platform, can be a barrier or avenue to promoting the health of such disadvantaged groups (Stone & Waldron, 2019). Older age has been associated with reduced e-health literacy in men, which can impose significant barriers for some males (Wills et al., 2020). In exploring the demographics of men who access e-health programs, and the relative benefits and barriers associated with specific end-user subgroups, important content and platform decisions and adjustments can be made (Oliffe, et al., 2020a; Soprovich et al., 2020).

In sum, evaluations of men’s e-health promotion programs, while encouraging, strongly support the need for additional evidence to map men’s health behavior changes and self-health promotion effects. The twofold purpose of the current study is to use a benchmark cohort as a reference group to: (1) describe associations between men’s usage levels of the e-health program Don’t Change Much (DCM) and their recent and intended health behavior changes, and (2) report an exploratory analysis of the moderating effects of demographic variables on the associations between DCM users and their recent and intended health behavior changes.

## Methods

By way of background, the DCM e-health program was launched by the Canadian Men’s Health Foundation in 2014 to inspire and equip men and their families to lead healthier lives. Accessible research-informed content is provided through quick tips and lobbying men’s strength-based actions to incrementally do the work of self-health. Text, video, and audio information is purposefully delivered to improve diet, exercise, sleep, and stress management as well as reduce alcohol use and/or smoking. The DCM content and delivery recognizes end-users as having diverse backgrounds, needs, and alignments to self-health, and the materials are purpose built to work with men wherever they are at, to advance their health promotion practices.

With approval from the University of British Columbia behavioral ethics review board, written consent was confirmed ahead of respondents providing demographic and self-reported survey questionnaire data detailing recent and intended health behavior changes. Data were collected from two Canadian male cohorts: (1) benchmark (reference group who had not accessed DCM), and (2) DCM users. Benchmark data collection procedures, and cross-sectional (Oliffe et al., 2020c; Flannigan et al., 2019; McCreary et al., 2020) and comparative findings (Oliffe et al., 2020a) have been published elsewhere. In brief, the 15-min benchmark questionnaire was administered April 20–28, 2017, via an online panel provider, and 2000 men, stratified by age and location based on Canadian census data, completed the survey. DCM users completed the same survey questionnaire between April 1, 2018 and March 31, 2019, providing details of their usage (duration and frequency) for each of the three DCM formats (web, newsletter, and social media). DCM users were recruited through pop-up ads on the DCM website and e-newsletter invitations and incentivized by the option to enter a $500 prize draw. DCM users comprised 3597 respondents, which was reduced to 1766 (1611 incomplete, 123 straight lining and/or speeding, 70 were not male, 19 resided outside of Canada, eight were under 19 years of age).

## Measures

Demographic data including age, education, employment, living arrangements (living with partner; children <19 years living at home), visible minority, sexual orientation, and household income (before taxes) were collected on the benchmark and DCM user cohorts. Respondents in both cohorts were also asked about recent and intended health behavior changes. To assess recent changes, respondents were asked “In the past 12 months, have you made any changes that would improve your health?” and invited to select all that applied from the following: (1) changed diet or improved eating habits, (2) made an effort to sit less and walk more, (3) increased exercise, sports, or physical activity, (4) drank less alcohol, (5) had a routine check-up or visit to doctor, (6) improved consistent sleep quality, (7) lost weight, (8) reduced stress level, (9) quit or reduced smoking. Intended changes data were collected through respondents selecting all that applied from the same nine items, with the stem question “In the next month (30 days), do you intend to make any changes that would improve your health?”

The work of Quinn and Chaudoir (2009) guided the conversion of categorical duration and frequency responses into continuous measures;

1) *When did you FIRST use, or subscribe to, the following resources?* Duration response options and continuous measure conversions: never before (0.0), in the past month (0.5), 1–6 months ago (3.5), 7–12 months ago (9.5), 13–24 months ago (18.5), more than 2 years ago (24.0), not sure (excluded).
2) *How often do you use or access the following resources?* Frequency response options and continuous measure conversions: several times a day (1095), once a day (365), several times a week (156), once a week (52), several times a month (36), once a month (12), several times a year (3), once a year (1), less often (.5), do not use (0), not sure (excluded).

The sum of the product of duration and frequency for each of the three DCM formats was used to calculate respondent exposure scores. For example, DCM users who responded “never before” (duration) and “do not use” (frequency) for all three DCM components were scored 0 and assigned to the limited exposure subgroup (*n* = 613, 34.7%). Respondents who used one DCM component “in the past month” (0.5 duration) “less often” (0.5 frequency) scored 0.25, and were classified low exposure (*n* = 826, 46.8%; range 0.25–680). Respondents who used two components, both “7–12 months ago” (9.5 duration) and “several times a month” (36 frequency) scored 684, and were classified high exposure (*n* = 327, 18.5%; range 684–30,570).

## Data Analysis

Cohen’s *d* was used to assess the magnitude of difference in means between two or more independent groups. In cases where the means of three or more independent groups were compared, partial eta-squared was computed first and then converted to Cohen’s *d* using formulae in Cohen (1988). To calculate an effect size for the strength of association between two categorical variables, Cramer’s V was used to compute values ranging from 0 to 1 (inclusive) (McHugh, 2013).

Logistic regression analysis was used to model the association between the level of exposure to DCM (Independent Variable (IV)) and recent and intended health behavior changes (Dependent Variable (DV)). R^2^ values were used to measure the proportion of variance in a DV that can be explained by the IVs in the regression models (Cohen, 1988). To analyze recent changes (in the past 12 months), the DVs were user specified behavior changes. For intended changes, the DVs were predicted behavior changes (in the next month). Linear regression models were used to evaluate the association between the number of recent and intended changes and level of exposure to DCM. The models computed the number of recent changes (DV) and the number of intended changes (DV). Benchmark respondents were the reference group, and DCM users were classified as limited, low, or high exposure. All logistic and linear regression models controlled for the following covariates: age, employment, living arrangements (lives with partner; children <19 years living at home), education, visible minority, sexual orientation, and household income (before taxes). Odds ratios indicated the strength of association between predictor variables and dichotomous outcome variables (Chen et al., 2010). Variance inflation factors (VIF) were computed as a collinearity diagnostic check.

Simple exploratory moderation analyses to assess for the interactions of DCM end-user demographics on associations between DCM exposure level and recent and intended health changes were conducted with PROCESS (Hayes, 2017). Separate moderation models were computed for all of the demographic variables. All variables were entered in one step, with simple moderation models comprising: (1) the main effect of low or high composite DCM exposure, relative to the benchmark reference group, (2) the demographic main effect, and (3) the interaction between DCM exposure and the specified demographic.

## Results

Benchmark respondents provided the reference group to compute the effects of DCM exposure on recent and intended health behavior changes. The magnitude of differences for demographic factors between benchmark respondents and DCM users indicated small to negligible effect sizes (Table 1). The majority of respondents in both cohorts were employed, lived with a partner, did not live with children under 19 years of age, had not graduated from university, and identified as heterosexual. Compared to benchmark respondents, a higher proportion of DCM users reported having a household income of $120,000 or more.

**Table 1.** Benchmark vs. DCM User Sample Demographics.

**Demographics**	Benchmark (*n* = 2000)	DCM^a^ Users (*n* = 1766)	χ^2^ (*df*)	*p* Value	Cramer’s V
**Age (mean, *SD*)**	46.99 (15.67)	50.08 (11.88)	6.869^b^ (*t*-test)	<.001	0.221^c^ (Cohen’s *d*)
**Employed (***n***, %)**			66.876 (1)	<.001	.133
Yes	1307 (65.4)	1368 (77.5)			
No	693 (34.7)	398 (22.5)			
**Living with a partner (***n***, %)**			34.412 (1)	<.001	.096
Yes	1210 (60.5)	1230 (69.6)			
No	790 (39.5)	536 (30.4)			
**Children <19 years old living with respondent (***n***, %)**			54.195 (1)	<.001	.120
Yes	441 (22.1)	578 (32.7)			
No	1559 (78)	1188 (67.3)			
**Highest level of education** (***n***, %)			2.101 (1)	.15	.024
Graduated university	823 (41.2)	768 (43.5)			
Other	1177 (58.9)	998 (56.5)			
**Visible minority (****n****, %)**			5.566 (1)	.02	.038
Yes	218 (10.9)	152 (8.6)			
No	1782 (89.1)	1614 (91.4)			
**Sexual orientation (*n*, %)**			15.848 (1)	<.001	.065
Heterosexual	1805 (90.3)	1520 (86.1)			
Gay, bisexual, questioning, other	195 (9.8)	246 (13.9)			
**Household income (*n*, %)**			95.598 (2)	<.001	.159
$59,999 or less	747 (37.4)	461 (26.1)			
$60,000–$119,999	855 (42.8)	730 (41.3)			
$120,000 or more	398 (19.9)	575 (32.6)			

a DCM = Don’t Change Much.

b As a ratio variable, the test performed was a *t*-test for this characteristic as indicated.

c As a ratio variable, the effect size computed was a Cohen’s *d* for this characteristic as indicated.

### Recent Health Behavior Changes

For the DCM group, all recent health behavior changes had significant associations with increased levels of exposure to DCM. Moderate effect sizes were observed in bivariate analyses between DCM exposure and “changed diet or improved eating habits” (χ^2^_3_ = 222, *p <* .001, Cramer’s V = 0.243) and “made an effort to sit less and walk more” (χ^2^_3_ = 160, *p <* .001, Cramer’s V = 0.206). Large effect sizes were observed in bivariate analyses between the total number of recent health changes and DCM high exposure level (Kruskal–Wallis *H* = 294, *p <* .001, Cramer’s V = 0.767). Compared to the benchmark cohort, high exposure respondents had significantly increased odds for all recent health behavior changes except “quit or reduced smoking” (OR = 0.920, 95% CI [0.606, 1.399]) while holding other predictor variables constant. Small effect sizes were observed for “made an effort to sit less and walk more” (OR = 2.996, 95% CI [2.347, 3.826]), “changed diet or improved eating habits” (OR = 2.879, 95% CI [2.252, 3.68]), and “improved consistent sleep” (OR = 2.655, 95% CI [2.05, 3.439]). Respondents with high exposure to DCM also had significantly greater total numbers of recent health changes while adjusting for covariates, with a small R^2^ effect size observed) (*F*_12,3753_ = 29.320, *p* < .001, R^2^ = 0.086) (Table 2).

**Table 2.** Logistic Regressions Between Demographics and Recent Health Behavior Changes.

	DCM Composite Level of Exposure (Ref = No exposure)					Household Composition					Household Income (Ref = $60,000–$119,999) (US$46,153.86–92,306.92)	
	Limited exposure	Low exposure	High exposure	Age (Years)	Employment	Lives with partner	Lives with children	Education	Visible minority	Sexual orientation	$59,999 or less(US$46,153.07 or less)	$120,000 or more (US$92,307.69 or more)
Changed diet or improved eating habits	2.022 (1.675, 2.44)***	2.867 (2.415, 3.405)***	2.879 (2.252, 3.68)***	1.009 (1.004, 1.015)**	1.152 (0.974, 1.363)	1.027 (0.872, 1.21)	0.957 (0.811, 1.129)	0.883 (0.767, 1.016)	1.449 (1.154, 1.82)**	1.063 (0.86, 1.313)	0.808 (0.683, 0.956)*	0.914 (0.771, 1.085)
Made an effort to sit less and walk more	1.532 (1.259, 1.864)***	2.341 (1.968, 2.785)***	2.996 (2.347, 3.826)***	1.014 (1.008, 1.02)***	0.941 (0.79, 1.119)	1.029 (0.869, 1.22)	0.972 (0.82, 1.153)	1.156 (1, 1.337)*	1.145 (0.904, 1.449)	1.029 (0.828, 1.28)	1.059 (0.889, 1.262)	1.143 (0.96, 1.361)
Increased exercise, sports, or physical activity	1.576 (1.307, 1.901)***	2.294 (1.934, 2.722)***	2.226 (1.744, 2.841)***	0.991 (0.986, 0.996)**	0.896 (0.76, 1.056)	1.078 (0.917, 1.267)	0.845 (0.718, 0.995)*	1.088 (0.948, 1.249)	1.311 (1.048, 1.642)*	0.913 (0.741, 1.125)	0.819 (0.695, 0.966)*	1.127 (0.953, 1.334)
Drank less alcohol	1.698 (1.38, 2.089)***	2.297 (1.914, 2.758)***	2.304 (1.785, 2.975)***	0.994 (0.988, 1)*	0.987 (0.822, 1.186)	1.072 (0.897, 1.282)	0.896 (0.748, 1.074)	0.755 (0.646, 0.881)***	0.883 (0.683, 1.143)	1.241 (0.993, 1.549)	0.912 (0.759, 1.095)	0.882 (0.731, 1.063)
Improved consistent sleep quality	1.755 (1.415, 2.177)***	2.033 (1.679, 2.462)***	2.655 (2.05, 3.439)***	1 (0.993, 1.006)	1.005 (0.83, 1.217)	0.845 (0.703, 1.016)	0.918 (0.759, 1.109)	1.007 (0.859, 1.182)	1.317 (1.027, 1.689)*	1.056 (0.836, 1.334)	0.946 (0.781, 1.145)	0.922 (0.759, 1.119)
Had a routine check-up or visit to doctor	1.627 (1.337, 1.98)***	1.573 (1.315, 1.882)***	1.826 (1.421, 2.347)***	1.046 (1.039, 1.052)***	0.667 (0.56, 0.793)***	1.084 (0.912, 1.287)	0.954 (0.803, 1.133)	0.925 (0.797, 1.072)	1.056 (0.825, 1.35)	1.106 (0.886, 1.382)	0.91 (0.761, 1.088)	1.07 (0.896, 1.277)
Lost weight	1.326 (1.089, 1.615)**	1.512 (1.269, 1.802)***	1.584 (1.238, 2.028)***	1.003 (0.997, 1.009)	1.102 (0.925, 1.314)	1.097 (0.925, 1.3)	0.932 (0.786, 1.105)	0.929 (0.803, 1.075)	1.025 (0.809, 1.3)	1.105 (0.89, 1.373)	0.858 (0.719, 1.023)	1.117 (0.939, 1.329)
Reduced stress level	1.41 (1.142, 1.741)**	1.533 (1.27, 1.851)***	1.875 (1.447, 2.429)***	0.998 (0.992, 1.004)	0.822 (0.684, 0.987)*	0.992 (0.828, 1.189)	0.98 (0.815, 1.179)	0.957 (0.819, 1.119)	1.064 (0.827, 1.37)	1.14 (0.907, 1.432)	0.965 (0.802, 1.161)	0.879 (0.726, 1.065)
Quit or reduced smoking	1.279 (0.967, 1.69)	0.936 (0.707, 1.24)	0.92 (0.606, 1.399)	0.989 (0.981, 0.997)**	1.255 (0.975, 1.617)	0.97 (0.76, 1.238)	0.775 (0.589, 1.021)	0.415 (0.325, 0.53)***	1.037 (0.725, 1.482)	1.322 (0.985, 1.774)	1.382 (1.086, 1.758)**	0.641 (0.468, 0.879)**

*Note*. DCM = Don’t Change Much.

* *p* < .05; **p < .01; ***p < .001; separate multiple logistic regressions were conducted for each outcome variable with all predictor variables entered on the same step.

### Intended Health Behavior Changes

For the DCM group, all intended health behavior changes had significant associations with increased levels of exposure to DCM. Moderate effect sizes were observed in bivariate analyses between DCM exposure and “improve consistent sleep quality” (χ^2^_3_ = 187, *p <* .001, Cramer’s V = 0.223), “change diet or improve eating habits” (χ^2^_3_ = 187, *p <* .001, Cramer’s V = 0.223), and “make an effort to sit less and walk more” (χ^2^_3_ = 166, *p <* .001, Cramer’s V = 0.210). Large effect sizes were observed in bivariate analyses between the total number of intended health changes and DCM exposure level (Kruskal–Wallis *H* = 338, *p <* .001, Cramer’s V = 0.845). Compared to the benchmark cohort, high exposure respondents had significantly increased odds for all of the intended health behavior changes except “Quit or reduce amount smoked” (OR = 1.368, 95% CI [0.889, 2.105]) while holding other predictor variables constant. Small effect sizes were observed for “reduce stress level” (OR = 3.428, 95% CI [2.643, 4.447]), “improve consistent sleep quality” (OR = 3.239, 95% CI [2.522, 4.161]), and “change diet or improve eating habits” (OR = 2.915, 95% CI [2.28, 3.727]). Respondents with high exposure to DCM also had significantly greater total numbers of intended health changes while adjusting for covariates, with a moderate R^2^ effect size observed (*F*_12,3753_ = 34.591, *p* < .001, R^2^ = 0.100) (Table 3).

**Table 3.** Logistic Regressions Between Demographics and Intended Health Behavior Changes.

	DCM Composite Level of Exposure (Ref = No exposure)					Household Composition					Household Income (Ref = $60,000–$119,999) (US$46,153.86–92,306.92)	
	Limited exposure	Low exposure	High exposure	Age (Years)	Employment	Lives with partner	Lives with children	Education	Visible minority	Sexual orientation	$59,999 or less (US$46,153.07 or less)	$120,000 or more (US$92,307.69 or more)
Improve consistent sleep quality	2.558 (2.093, 3.127)***	2.847 (2.376, 3.41)***	3.239 (2.522, 4.161)***	0.99 (0.984, 0.996)**	1.164 (0.97, 1.397)	0.963 (0.808, 1.147)	0.995 (0.834, 1.186)	0.944 (0.812, 1.098)	1.387 (1.095, 1.756)**	1.054 (0.844, 1.316)	0.99 (0.826, 1.185)	0.826 (0.688, 0.993)*
Change diet or improve eating habits	2.881 (2.374, 3.497)***	2.413 (2.025, 2.876)***	2.915 (2.28, 3.727)***	0.992 (0.986, 0.997)**	1.022 (0.858, 1.218)	1.089 (0.918, 1.291)	1.195 (1.009, 1.416)*	0.927 (0.801, 1.073)	1.326 (1.052, 1.67)*	1.106 (0.891, 1.373)	1.041 (0.875, 1.24)	0.837 (0.701, 0.999)*
Make an effort to sit less and walk more	2.141 (1.763, 2.598)***	2.467 (2.069, 2.94)***	2.79 (2.184, 3.564)***	1.013 (1.008, 1.019)***	0.85 (0.714, 1.011)	0.93 (0.784, 1.102)	1.182 (0.996, 1.402)	0.9 (0.777, 1.042)	1.06 (0.834, 1.348)	1.088 (0.875, 1.353)	1.029 (0.864, 1.226)	0.946 (0.792, 1.129)
Reduce stress level	2.361 (1.907, 2.924)***	2.54 (2.099, 3.074)***	3.428 (2.643, 4.447)***	0.979 (0.973, 0.985)***	1.124 (0.925, 1.365)	0.994 (0.826, 1.196)	0.998 (0.83, 1.201)	0.783 (0.667, 0.92)**	1.324 (1.035, 1.694)*	1.221 (0.972, 1.535)	0.865 (0.714, 1.047)	0.878 (0.724, 1.064)
Increase exercise, sports, or physical activity	1.728 (1.432, 2.086)***	2.088 (1.76, 2.477)***	2.385 (1.861, 3.058)***	0.989 (0.984, 0.995)***	1.041 (0.883, 1.226)	1.197 (1.019, 1.407)*	1.164 (0.989, 1.371)	1.018 (0.887, 1.17)	1.275 (1.018, 1.598)*	1.179 (0.957, 1.453)	0.987 (0.837, 1.163)	0.888 (0.75, 1.051)
Lose weight	1.867 (1.547, 2.252)***	1.783 (1.506, 2.11)***	1.439 (1.132, 1.831)**	1.009 (1.003, 1.014)**	1.215 (1.028, 1.436)*	1.16 (0.986, 1.364)	1.206 (1.025, 1.419)*	0.858 (0.746, 0.986)*	0.935 (0.745, 1.174)	1.199 (0.973, 1.477)	0.985 (0.834, 1.163)	1.065 (0.9, 1.26)
Drink less alcohol	1.869 (1.477, 2.367)***	2.291 (1.859, 2.823)***	2.857 (2.162, 3.777)***	0.996 (0.989, 1.003)	1.091 (0.883, 1.348)	0.909 (0.742, 1.113)	0.892 (0.726, 1.097)	0.669 (0.559, 0.8)***	1.178 (0.889, 1.561)	1.014 (0.784, 1.311)	0.917 (0.742, 1.132)	1.025 (0.83, 1.266)
Have a routine check-up or visit to doctor	1.872 (1.503, 2.33)***	1.669 (1.358, 2.05)***	1.911 (1.447, 2.524)***	1.019 (1.013, 1.026)***	0.951 (0.779, 1.162)	0.875 (0.72, 1.063)	1.117 (0.915, 1.364)	0.883 (0.745, 1.047)	1.31 (0.999, 1.718)	1.142 (0.891, 1.463)	0.951 (0.779, 1.161)	0.731 (0.592, 0.902)**
Quit or reduce smoking	1.261 (0.913, 1.742)	1.002 (0.726, 1.383)	1.368 (0.889, 2.105)	0.985 (0.976, 0.994)**	1.179 (0.886, 1.567)	0.656 (0.498, 0.865)**	0.947 (0.693, 1.296)	0.366 (0.274, 0.489)***	0.695 (0.441, 1.096)	1.207 (0.864, 1.687)	1.239 (0.944, 1.627)	0.597 (0.411, 0.868)**

*Note*. DCM = Don’t Change Much.

* *p* < .05; **p < .01; ***p < .001; separate multiple logistic regressions were conducted for each outcome variable with all predictor variables entered on the same step.

### Moderating Effects of Demographics

An exploratory moderation analysis indicated age, employment, living arrangements, education, and income had an interaction effect on associations between DCM exposure and four recent health behavior changes: “changed diet or improved eating habits,” “made an effort to sit less and walk more,” “had a routine check-up or visit to doctor,” and “reduced stress level.” Among these, higher education levels (graduated university) had an enhancing moderator effect on the association between DCM exposure and recent “changes to diet or improved eating habits” (OR = 1.3, 95% CI [1.027, 1.884]) and “made an effort to sit less and walk more” (OR = 1.434, 95% CI [1.056, 1.949]) (please see Table 4). Interaction effects of age, employment, education, and income were found for five intended health behavior changes. Models for the number of intended health behavior changes indicated younger age (R^2^ = .095, *F*(110.610), *p* = .001, *b* = −0.023), being employed (R^2^ = .092, *F*(106.80), *p* = .001, *b* = 0.442), and annual household income <$120,000 (R^2^ = .093, *F*(107.742), *p* = .001, *b* = −0.463) accounted for a significant proportion of the variance for intended health behavior changes (Table 5).

**Table 4.** Analysis of Statistically Significant Moderating Effects Between Demographics and Recent Health Changes.

Predictor Variables	Outcome Variables			
	OR (95% CI)			
	Changed diet or improved eating habits	Made an effort to sit less and walk more	Had a routine check-up or visit to doctor	Reduced stress level
DCM^a^		5.829 (3.225, 10.534)***		
Age		1.020 (1.014, 1.027)***		
DCM x Age		0.983 (0.972, 0.995)**		
DCM			1.256 (0.936, 1.686)	
Employment			0.339 (0.279, 0.411)***	
DCM x Employment			1.738 (1.230, 2.455)**	
DCM			2.000 (1.525, 2.624)***	
Lives with partner			1.747 (1.436, 2.125)***	
DCM x Lives with partner			0.691 (0.499, 0.957)*	
DCM				1.766 (1.455, 2.145)***
Lives with children				1.137 (0.886, 1.461)
DCM x Lives with children				0.643 (0.445, 0.930)*
DCM	2.579 (2.106, 3.160)***	2.172 (1.766, 2.671)***		
Education	0.774 (0.641, 0.936)**	0.969 (0.793, 1.184)		
DCM x Education	1.391 (1.027, 1.884)*	1.434 (1.056, 1.949)*		
DCM		2.864 (2.387, 3.435)***		
$120,000 or more		1.381 (1.088, 1.751)**		
DCM x $120,000 or more		0.656 (0.468, 0.922)*		

*Note*. DCM = Don’t Change Much.

a Dichotomous variable, with 1 = low or high composite exposure, and 0 = benchmark.

* *p* < .05; **p < .01; ***p < .001. Separate moderation models were computed for all nine recent health changes and all demographic variables (age, employment, lives with partner, lives with children, education, visible minority, sexual orientation, $59,999 or less, $120,000 or more). Models were also computed for the number of recent health changes and all demographics. Models with statistically insignificant moderator effects were omitted from display in the table.

**Table 5.** Analysis of Statistically Significant Moderating Effects Between Demographics and Intended Health Changes.

Predictor Variables	Outcome Variables								
	OR (95% CI)					*B*	F	*p*	R^2^
	Change diet or improve eating habits	Make an effort to sit less and walk more	Increase exercise, sports, or physical activity	Have a routine check-up or visit to doctor	Quit or reduce smoking	Number of intended health changes			
DCM^a^	5.051 (2.804, 9.096)***	5.498 (3.029, 9.982)***	4.535 (2.506, 8.204)***	5.045 (2.493, 10.209)***		2.465***	110.610	<.001	.095
Age	0.995 (0.989, 1.002)	1.019 (1.013, 1.026)***	0.993 (0.988, 0.999)*	1.025 (1.017, 1.033)***		0.001			
DCM x Age	0.986 (0.974, 0.997)*	0.984 (0.972, 0.995)**	0.986 (0.974, 0.997)*	0.978 (0.965, 0.991)**		−0.023***			
DCM	1.828 (1.339, 2.495)***	1.924 (1.426, 2.595)***	1.616 (1.204, 2.170)**			1.002***	106.80	<.001	.092
Employment	1.016 (0.822, 1.256)	0.627 (0.510, 0.771)***	1.086 (0.902, 1.308)			−0.002			
DCM x Employment	1.454 (1.015, 2.082)*	1.549 (1.090, 2.202)*	1.437 (1.020, 2.024)*			0.442*			
DCM					1.085 (0.798, 1.475)				
Education					0.436 (0.306, 0.623)***				
DCM x Education					0.488 (0.252, 0.944)*				
DCM		2.290 (1.909, 2.748)***							
$59,999 or less		0.898 (0.728, 1.107)							
DCM x $59,999 or less		1.430 (1.011, 2.025)*							
DCM		2.912 (2.425, 3.497)***		1.956 (1.590, 2.406)***		1.507***	107.742	<.001	.093
$120,000 or more		1.242 (0.972, 1.586)		0.913 (0.675, 1.236)		0.038			
DCM x $120,000 or more		0.605 (0.428, 0.855)**		0.652 (0.427, 0.996)*		−0.463**			

*Note*. DCM = Don’t Change Much.

a Dichotomous variable, with 1 = low or high composite exposure, and 0 = benchmark.

* *p* < .05; **p < .01; ***p < .001. Separate moderation models were computed for all nine intended health changes and all demographic variables (age, employment, lives with partner, lives with children, education, visible minority, sexual orientation, $59,999 or less, $120,000 or more). Models were also computed for the number of intended health changes and all demographics. Models with statistically insignificant moderator effects were omitted from display in the table.

## Discussion and Conclusion

The current study findings confirm and extend our previous work (Oliffe et al., 2020a), and by extension affirm the strong potential of men’s e-health promotion programs (Robinson & Robertson, 2010). Though not claiming attribution, the statistically significant positive associations between men’s DCM exposure levels and their recent and intended health behavior changes warrant discussion of three key issues as a means to scoping potential DCM adjustments, and offering recommendations to advance men’s e-health promotion work.

First, in describing specific recent and intended health behavior changes made available are opportunities to consider adjustments to DCM. Recent changes in “changed diet or improved eating habits” and “made an effort to sit less and walk more” reflect, at least in part, the appeal of DCM’s content and the lobby for men to make incremental and proportion-based adjustments (<sitting and >walking; balancing food groups). Within these contexts, the call to action is not wholesale change or abstinence; instead, DCM focuses on strategies for making adjustments (getting more steps and cooking [and eating] a variety of foods). That “reduce stress” and “improve consistent sleep quality” featured prominently as intended health behavior changes along with reports of recent “improved consistent sleep quality” underscores the importance building on the tailored DCM stress reduction and sleep-aiding resources. A review of interventions to promote sleep health in men (Soprovich et al., 2020) reported moderate level evidence for sleep health programs incorporating physical activity and stress management components. With this in mind, there may be traction for bundling some DCM content to engage men with interlocking strategies for concurrently reducing stress and increasing physical activity and sleep quality. Due diligence also includes attention to health behaviors drawing relatively little change. Specifically, the respondents’ low recent and intended changes for “quit or reduce smoking” likely reflect the small subpopulation of male smokers in Canada—and the fewer DCM end-users who smoke. In addition, because men’s e-health tobacco reduction and smoking cessation (TRSC) resources tend to be specialized (Bottorff et al., 2016), it is unlikely that DCM end-users who smoke arrived with the goal of TRSC.

Second, while e-health resources engage many men, the issues of reach and effectiveness continue to challenge the field. That the current study regression findings held when controlling across key demographics synonymous with social determinants of health (i.e., income, employment, education etc.) suggests DCM is accessible to, and engaging of, men from diverse backgrounds. That said, the results of the moderation analysis reveal enhancing interactions for younger, employed men, with an annual household income <$120,000. One possibility is that the DCM resources resonate most for men with those demographics. Of course, there is the possibility that these subgroups are also more motivated to make health changes. One challenge related to this finding is how best to sustain end-users drawing the biggest benefits while building content to grow those gains in other subgroups.

Third, while direct comparisons with previous DCM findings (Oliffe et al., 2020a) were not the aim of the current study, it is important to note that there were subtle changes to behavior change rankings and the strength of the associations with DCM exposure. For most end-users’ recent and intended health behavior changes, the odds ratios were lower (though significant) in the current study compared to the previous study results. One explanation for this is that the earlier study (Oliffe et al., 2020a) reported data collected January 1 through March 31 2018—a new-year period strongly linked to resolutions including health behavior changes. The current study data were collected over a 1-year period (April 1, 2018 through March 31, 2019), and this may have flattened the potential new-year peak reported in the previous study. Though speculation, this explanation should remind us that year-end through early new-year campaigns can be especially timely for engaging men with e-health promotion.

The high potential for familywise errors in conducting numerous separate regression analyses is a methodological limitation of the current study. Self-report biases for respondents’ DCM usage and recent and intended health behavior changes are limitations, as is the cross-sectional study design. These limitations can however be addressed by including qualitative interviews to further contextualize and augment statistical results. The current study design also underscores the need for longitudinal research to evaluate change more fully over time.

Within diverse platform delivery, fidelity, and focus, there is a pressing need to advance evaluative evidence in men’s e-health promotion. The tensions between reporting associations amid wanting to claim attribution might best be resolved with some concessions that men’s health behaviors shift over time and context in response to an array of influences. This is not to deny the need for some control trials to prove effect; rather, it is an acknowledgment that men’s e-health promotion programs can (and often do) assist men to advance their health, and by extension, the well-being of their families.
